# Inhibitory effect of *Newtonia* extracts and myricetin-3-*o*-rhamnoside (myricitrin) on bacterial biofilm formation

**DOI:** 10.1186/s12906-020-03139-4

**Published:** 2020-11-23

**Authors:** Katlego E. Motlhatlego, Muna Ali Abdalla, Carmen M. Leonard, Jacobus N. Eloff, Lyndy J. McGaw

**Affiliations:** 1 Phytomedicine Programme, Department of Paraclinical Sciences, Faculty of Veterinary Science, University of Pretoria, Private Bag X04, Onderstepoort, 0110 South Africa; 2 Current address: Department of Pharmacy and Pharmacology, Faculty of Health Sciences, University of the Witwatersrand, 7 York Road, Parktown, Johannesburg, 2193 South Africa; 3 Department of Food Science and Technology, Faculty of Agriculture, University of Khartoum, 13314 Khartoum North, Sudan; 4 Microbiology Laboratory, Department of Pharmaceutical Sciences, Tshwane University of Technology, Private Bag X680, Pretoria, 0001 South Africa

**Keywords:** *Newtonia*, Fabaceae, Diarrhoea, Antimicrobial, Cytotoxicity, Biofilm formation

## Abstract

**Background:**

Diarrhoea is a major health issue in both humans and animals and may be caused by bacterial, viral and fungal infections. Previous studies highlighted excellent activity of *Newtonia buchananii* and *N. hildebrandtii* leaf extracts against bacterial and fungal organisms related to diarrhoea-causing pathogens. The aim of this study was to isolate the compound(s) responsible for antimicrobial activity and to investigate efficacy of the extracts and purified compound against bacterial biofilms.

**Methods:**

The acetone extract of *N. buchananii* leaf powder was separated by solvent-solvent partitioning into eight fractions, followed by bioassay-guided fractionation for isolation of antimicrobial compounds. Antibacterial activity testing was performed using a broth microdilution assay. The cytotoxicity was evaluated against Vero cells using a colorimetric MTT assay. A crystal violet method was employed to test the inhibitory effect of acetone, methanol: dichloromethane and water (cold and hot) extracts of *N. buchananii* and *N. hildebrandtii* leaves and the purified compound on biofilm formation of *Pseudomonas aeruginosa*, *Escherichia coli*, *Salmonella* Typhimurium, *Enterococcus faecalis*, *Staphylococcus aureus* and *Bacillus cereus*.

**Results:**

Myricetin-3-*o*-rhamnoside (myricitrin) was isolated for the first time from *N. buchananii*. Myricitrin was active against *B. cereus*, *E. coli* and *S. aureus* (MIC = 62.5 μg/ml in all cases). Additionally, myricitrin had relatively low cytotoxicity with IC_50_ = 104 μg/ml. Extracts of both plant species had stronger biofilm inhibitory activity against Gram-positive than Gram-negative bacteria. The most sensitive bacterial strains were *E. faecalis* and *S. aureus*. The cold and hot water leaf extracts of *N. buchananii* had antibacterial activity and were relatively non-cytotoxic with selectivity index values of 1.98–11.44.

**Conclusions:**

The purified compound, myricitrin, contributed to the activity of *N. buchananii* but it is likely that synergistic effects play a role in the antibacterial and antibiofilm efficacy of the plant extract. The cold and hot water leaf extracts of *N. buchananii* may be developed as potential antibacterial and antibiofilm agents in the natural treatment of gastrointestinal disorders including diarrhoea in both human and veterinary medicine.

## Background

Diarrhoea is a neglected disease responsible for over 700,000 deaths annually of children under the age of five worldwide [1]. Ciprofloxacin has been used for the treatment of gastrointestinal infections, such as diarrhoea, for decades [2]. The over-prescribing and incorrect use of antibiotics commonly used to treat diarrhoea and other infections have led to global antibiotic resistance against several microbes. This has greatly impacted on the efficacy of most available antibacterial drugs [3]. Antibiotics are less effective when biofilms form because of the relative impermeability of biofilms, the variable physiological status of microorganisms, subpopulations of persistent strains, the presence of variations of phenotypes and the expression of genes involved in the general stress response [4]. Consequently, biofilms are a recognized source of recurrent, persistent or sporadic bacterial infections [5, 6].

The treatment of infection has become difficult because the biofilm mode of microbial growth has increased the survival strategies and resistance levels of microbes to drugs [3].

Many drugs have been developed that were originally sourced from natural products [7]. Plants contain a variety of secondary metabolites such as alkaloids, flavonoids, glycosides, phenols, saponins, steroids, terpenoids and tannins [8]. These metabolites may have individual bioactivity, or may act together synergistically to disrupt growth or pathogenic pathways of disease-causing organisms [9].

Some natural products are known to inhibit biofilm formation or preformed biofilms [3]. Plants are therefore a potential source of novel antibiofilm agents [4, 10, 11] worthy of further investigation.

A wide variety of medicinal plants are used in southern Africa to treat gastrointestinal ailments as well as other infections. Two of these species include *Newtonia hildebrandtii* (Vatke) Torre and *Newtonia buchananii* (Baker) G.C.C. Gilbert & Boutiqu of the family Fabaceae, which are used for the treatment of skin conditions and wounds and for an upset stomach [12, 13]. In a previous study, the acetone and dichloromethane:methanol (1:1) extracts of the leaves and stems of the two species had very good antibacterial activity against a range of bacterial species with minimum inhibitory concentration (MIC) values as low as 0.02 mg/ml [14]. This motivated the present study where the isolation of compounds responsible for this promising activity was undertaken. Additionally, hot and cold water extracts of the two species were included to more closely replicate the traditional methods of preparation and to provide evidence to support their use in traditional medicine against stomach upsets. As biofilms are an important mechanism employed by bacteria to establish infection and avoid antibiotic activity, the organic and aqueous extracts as well as the purified compound were investigated for their ability to inhibit biofilm formation.

## Methods

### Plant material and extraction

The plant species were collected from labelled trees in the Lowveld National Botanical Garden in Nelspruit, Mpumalanga, South Africa in December 2014. Voucher specimens (PRU 122347 for *N. hildebrandtii* and PRU 122348 for *N. buchananii*) were prepared and lodged in the H.G.W.J. Schweickerdt Herbarium at the University of Pretoria (South Africa) for reference purposes.

The collected plant material was dried at room temperature in a well-ventilated room and ground to a fine powder in a Macsalab Mill (Model 2000 LAB Eriez). One gram of each plant part (leaves and stems, and in the case of *N. buchananii* also the seeds and seedpods combined) was separately extracted in 10 ml of acetone, 1:1 MeOH-DCM (technical grade, Merck), cold distilled water, or boiling distilled water in polyester centrifuge tubes. The tubes were vigorously shaken for 30 min on an orbital shaker, then centrifuged at 4000 x g for 10 min. The supernatant was filtered through Whatman No.1 filter paper before it was transferred into pre-weighed glass containers. This was repeated thrice on the same plant material and the solvent was removed by evaporation under a stream of air in a fume hood at room temperature to yield the dried crude extract. For isolation purposes, a similar extraction method was followed where 300 g of *N. buchananii* leaf material was extracted in 3000 ml of acetone (technical grade, Merck) in a 5 L glass bottle. The bottle was vigorously shaken and left overnight and the supernatant was filtered through Whatman No.1 filter paper before it was transferred into pre-weighed glass containers. The extraction yield was calculated as follows:

$$ \mathrm{Plant}\ \mathrm{crude}\ \mathrm{extract}\mathrm{ion}\ \mathrm{yield}\ \left(\%\right)=\frac{\mathrm{Mass}\ \mathrm{of}\ \mathrm{dried}\ \mathrm{extract}\ \left(\mathrm{g}\right)\ }{\mathrm{Mass}\ \mathrm{of}\ \mathrm{plant}\ \mathrm{powder}\ \mathrm{extract}\mathrm{ed}\ \left(\mathrm{g}\right)}\times 100 $$

### Antimicrobial assay

The antimicrobial activity of the water extracts was determined against the following bacteria: *Staphylococcus aureus* (ATCC 29213), *Bacillus cereus* (ATCC 21366), *Enterococcus faecalis* (ATCC 29212), *Escherichia coli* (ATCC 25922), *Pseudomonas aeruginosa* (ATCC 27853) and *Salmonella enterica subsp. enterica* serovar Typhimurium (ATCC 39183). The antimicrobial activity was evaluated in terms of minimum inhibitory concentration (MIC) using a rapid broth microdilution technique with *p*-iodonitrotetrazolium violet (INT) as a growth indicator [15]. The INT dissolved in hot water (40 μl of a 0.2 mg/ml stock solution) was added to the wells and incubated at 37 °C for an hour. The MIC values were recorded as the lowest concentration of the extracts that inhibited bacterial growth, as indicated by a marked reduction in colour formation. The *p*-iodonitrotetrazolium violet turns to a red-pink formazan where bacterial growth is not inhibited. The assays were repeated three times with three replicates in each assay.

### Cytotoxicity assay

The cytotoxicity of the aqueous extracts against African green monkey (Vero) kidney cells was determined by the MTT [3-(4,5-dimethylthiazol-2-yl)-2,5-diphenyltetrazolium bromide] reduction assay as previously described by Mosmann (1983) [16] with slight modifications. Vero cells were maintained at 37 °C and 5% CO_2_ in a humidified environment in Minimal Essential Medium (MEM) containing L-glutamine (Lonza, Belgium) and supplemented with 5% fetal bovine serum (Capricorn Scientific Gmbh, South America) and 1% gentamicin (Virbac, RSA). These cells were seeded at a density of 10^5^ cells/ml (100 μl) in 96-well microtitre plates and incubated at 37 °C overnight to allow attachment. After incubation, extracts (100 μl) at varying final concentrations were added to the wells containing cells. Doxorubicin hydrochloride (Pfizer) was used as a positive control. Wells made of cells in fresh medium without treatment and a blank containing only the fresh medium were used as negative controls. The plates were further incubated at 37 °C and 5% CO_2_ for 48 h. After incubation, the medium was aspirated from the cells, which were then washed with phosphate-buffered saline (PBS). Then, 200 μl fresh medium together with 30 μl MTT (5 mg/ml in PBS) were added to each well and the plates were incubated at 37 °C in a 5% CO_2_ humidified incubator for 4 h. The medium was carefully aspirated from the wells and the formed formazan crystals were dissolved in dimethylsulfoxide (DMSO). The plates were placed on an orbital shaker for about 2 min. The absorbance was measured on a microplate reader (BioTek Synergy) at 570 nm. Cell growth inhibition for each extract was expressed in terms of LC_50_ values, defined as the lethal concentration that caused 50% inhibition of cell viability. The selectivity index (SI) values were calculated by dividing LC_50_ values by the MIC values in the same units (SI = LC_50_/MIC). Tests were carried out in quadruplicate and each experiment was repeated thrice.

### Isolation of the compound

*N. buchananii* was selected for isolation of antimicrobial compounds owing to the high antimicrobial activity of the crude extracts. The crude acetone extract (43.8 g) of *N. buchananii* was subjected to silica gel column chromatography (7.5 × 60 cm), silica gel 60: 0.05–0.2 mm, 70–270 mesh (Macherey-Nagel & Co) eluted with CH_2_Cl_2_ followed by stepwise addition of CH_3_OH (gradient 0 to 100%) to yield eight fractions. The antibacterial activity of the eight fractions was determined against the six bacterial pathogens known to cause diarrhoea. The next step was to isolate the bioactive compounds from the most active fraction with low cytotoxicity using Sephadex LH-20 column chromatography.

### Structure elucidation of the isolated compound

The compound was characterized by means of 1D and 2D NMR (spectroscopic and mass spectrometry analysis. ^1^H NMR and 2D NMR including COSY, HMQC, and HMBC data were acquired on a 400 MHz NMR spectrometer (Bruker Avance III 400 MHz). HPLC-HR-ESI–MS was performed on Waters Acquity Ultra Performance Liquid Chromatography (UPLC®) system hyphenated to a quadrupole-time-of-flight (QTOF) instrument.

### Inhibition of biofilm formation

The biofilm inhibition assay was determined according to Sandasi et al. (2011) [10]. In this study the various stages of biofilm development were assumed to be: no attachment/planktonic (T0), initial attachment (T4), irreversible attachment (T24) and mature biofilm (T48). The extracts were resuspended in acetone for the acetone and MeOH: DCM extracts, and sterile distilled water for water extracts and prepared to the same concentration as the MIC value ( [14], Table 1). Briefly, 100 μl aliquots of plant extracts or compound were placed into wells of a 96 well microtitre plate to prevent initial attachment. A 100 μl aliquot of standardised cultures (OD_560_ = 0.02 equivalent to 1.0 × 10^6^ CFU/ml) of *P. aeruginosa*, *S.* Typhimurium, *S. aureus*, *E. faecalis*, *E. coli* or *B. cereus* was added into the wells and incubated (Scientific Group) at 37 °C for 0, 4, 24 and 48 h (i.e. T0, T4, T24 and T48) respectively without shaking. After the selected incubation periods (T0, T4, T24 and T48), 100 μl of the extracts and the isolated compound (at the MIC values) were added at the different biofilm development stages and incubated at 37 °C for 24 h.

**Table 1:** Antibacterial activity and cytotoxicity of water extracts of two *Newtonia* species

Plant name	Plant part	Extraction yield (%)	Extract	MIC in mg/ml and test organisms						LC_50_ (mg/ml)
				Ec	Bc	Ef	Pa	St	Sa	
*Newtonia hildebrandtii*	Leaves	27.5	Cold water	0.31	0.31	0.78	0.63	0.52	1.25	2.64
		32.00	Hot water	0.31	0.31	0.78	0.63	0.63	1.25	2.47
*Newtonia buchananii*	Leaves	11.25	Cold water	0.84	0.31	0.78	1.25	0.63	1.25	2.48
		13.67	Hot water	0.63	0.16	0.78	0.63	0.63	0.63	2.95
Ciprofloxacin (MIC in ug/ml)				1.56	0.78	0.000098	0.00156	0.0063	0.0063	

Minimum Inhibitory Concentration (MIC); concentration lethal to 50% of the cells (LC_50_). *Escherichia coli* (Ec), *Bacillus cereus* (Bc), *Enterococcus faecalis* (Ef), *Pseudomonas aeruginosa* (Pa), *Salmonella* Typhimurium (St) and *Staphylococcus aureus* (Sa)

Ciprofloxacin at a concentration of 0.01 mg/ml served as a positive control for all organisms used in this study. Acetone or sterile water with bacterial cells served as negative controls. As soon as the selected incubation periods (T0, T4, T24 and T48) ended, the biofilm biomass was assayed using the modified crystal violet (CV) staining assay [17]. Briefly, the microtitre plates were washed three times with sterile distilled water and allowed to air-dry. Following this the plates were oven-dried at 60 °C for 45 min. The wells were then stained with 100 μl of 1% crystal violet and incubated at room temperature for 15 min after which the plates were washed three times with sterile distilled water to remove unabsorbed stain. The semi-quantitative assessment of biofilm formation was performed by adding 125 μl of ethanol to de-stain the wells. A 100 μl aliquot of the de-staining solution was transferred to a new plate and the absorbance was measured with SoftMax Pro 6 at 590 nm using a microplate reader (SpectraMax M2). The experiments were performed in triplicate for each extract or compound and mean absorbance of 8 replicates for each experiment calculated. The mean absorbance of the samples was determined, and percentage inhibition calculated using the equation described below:

$$ \mathrm{Percentage}\ \mathrm{inhibition}=\left({\mathrm{OD}}_{\mathrm{Negative}\ \mathrm{control}}\kern0.5em -\kern0.5em {\mathrm{OD}}_{\mathrm{Experimental}}\times 100\right)/{\mathrm{OD}}_{\mathrm{Negative}\ \mathrm{control}} $$

### Statistical analysis

Statistical analysis was conducted with GraphPad InStat Software and results were compared using the StudentNewman Keuls and Dunnett‘s tests. Data were analysed using a one-way analysis of variance to compare within each species, where there were significant differences, a Duncans Multiple Range Post Hoc test was used to separate the means. Results were considered significantly different when *P*< 0.05.

## Results

### Minimum inhibitory concentration and cytotoxicity of the water extracts

The water extracts of *N. hildebrandtii* and *N. buchananii* had some antibacterial activity (Table 1). Although the best antibacterial effect of both water leaf extracts was against *B. cereus* with MIC values of 0.31 mg/ml, the hot water leaf extract of *N. buchananii* had an MIC value of 0.16 mg/ml. However this was not noteworthy as extracts with MIC values above 100 μg/ml are considered to have relatively low antibacterial activity [18]. Importantly, the extracts had SI values above 1 and as high as 18.44 (Table 2). This means that the water extracts of both *Newtonia* species were more toxic to the microorganisms than against mammalian Vero cells which is very promising.

**Table 2:** Selectivity index (SI) of water extracts of the selected *Newtonia* species

Plant name	Plant part	Extract	SI = LC_50_/MIC					
			Ec	Bc	Ef	Pa	St	Sa
*N. hildebrandtii*	Leaves	Cold water	**8.52**	**8.52**	**3.39**	**4.19**	**5.08**	2.11
		Hot water	**7.97**	**7.97**	**3.17**	**3.92**	**3.92**	1.98
*N. buchananii*	Leaves	Cold water	2.95	**8.00**	**3.18**	1.98	**3.93**	1.98
		Hot water	**4.68**	**18.44**	**3.78**	**4.68**	**4.68**	**4.68**

Selectivity Index (SI); *Escherichia coli* (Ec), *Bacillus cereus* (Bc), *Enterococcus faecalis* (Ef), *Pseudomonas aeruginosa* (Pa), *Salmonella typhimurium* (St) and *Staphylococcus aureus* (Sa). Values in bold highlight promising SI values

### Characterization of the isolated compound

The compound was isolated as a yellow powder, which produced a strong ultraviolet (UV) absorbing band on TLC at 257 nm and turned to yellow with vanillin-sulphuric acid spray reagent. The ESI-HRMS afforded the molecular formula as C_20_H_21_O_12_. The molecular weight was determined by ESI-MS (*m/z* 463.0907 [M*-*H]^−^, 927.1844 [2 M*-*H]^−^). ^1^H and ^13^C NMR spectra indicated the presence of a flavonol rhamnoside. The ^1^H NMR and ^13^C NMR data are presented in Table 3.

**Table 3:** NMR data of myricetin-3-*O-*rhamnoside (myricitrin, in CD_3_OD) and literature data (in DMSO-d6, 500 Hz)

Position	Myricitrin		Literature data (Kassem et al., 2016) [19] in (DMSO-*d*_6_, 500 Hz)	
	H	C	H	C
2		156.4		157.0
3		137.0		134.7
4		181.4		178.1
5		165.3		161.7
6	6.21, d, *J* = 2.0 Hz	99.5	6.20, d, *J* = 2.0 Hz	99.1
7		165.7		165.6
8	6.38, d, *J* = 2.0 Hz	94.3	6.3, d, *J* = 2.5 Hz	94.1
9		158.5		157.8
10		105.7		104.2
1′		123.1		120.1
2′, 6′	6.96, s	109.6	6.89, s	108.4
3′, 5′		145.7		146.3
4′		136.4		137.0
1″	5.32, brd s, *J* = 1.5 Hz	102.0	NA	102.4
2″	4.23, dd, *J* = 1.6, 3.3 Hz	71.9	3.1–4.0, m	71.8
3″	3.79, dd, *J* = 3.6, 9.6 Hz	71.9		70.9
4″	3.37, d, *J* = 9.6 Hz	73.1		71.0
5″	3.53, dd, *J* = 6.3, 9.6 Hz	71.9		70.4
6″	0.96, d, *J* = 6.3 Hz	17.1	0.77, d, *J* = 6 Hz	18.0

### Minimum inhibitory concentration and cytotoxicity of the isolated compound

The antimicrobial activity of an isolated compound is generally considered significant if the MIC is 10 μg/ml or lower, moderate if MIC is between 10 and 100 μg/ml and low if MIC is greater than 100 μg/ml [18, 20, 21]. In this study it was found that myricitrin (as presented in Table 4) showed moderate activity at a MIC of 62.5 μg/ml against *E. coli*, *S. aureus* and *B. cereus*, additionally it was relatively non-toxic with an LD_50_ of 104 μg/ml.

**Table 4:** Antimicrobial activity and cytotoxicity of myricitrin from *Newtonia buchananii*

Test substance	MIC (μg/ml) and test organisms						LC_50_ (μg/ml)	Selectivity index (SI) and test organisms SI = LC_50_/MIC					
	Ec	Bc	Ef	Pa	St	Sa		Ec	Bc	Ef	Pa	St	Sa
Myricitrin	62.5	62.5	250	250	125	62.5	104.42	1.67	1.67	0.42	0.42	0.83	1.67
Gentamicin	1.56 × 10^−3^	1.56 × 10^−3^	9.8 × 10^−5^	7.8 × 10^−4^	1.5610^−3^	7.8 × 10^−4^							

### Anti-biofilm potential of the extracts and the compound

The anti-biofilm activity of the *Newtonia* extracts, the antibacterial compound myricitrin, and the control was determined (Fig. 2). The graphs represent the biofilm inhibitory activity (BIA) of the crude extracts against some human pathogens known to cause diarrhoea.

The Gram-negative organism, *P. aeruginosa,* is a model biofilm-forming organism and BIA was evaluated at different time intervals (Fig. 2a). At time 0 h (organism in planktonic form) the cold water extracts of both plants enhanced the growth or biofilm development of the test organism. This enhancement was also observed at the 4 and 24 h timeframe and then poor inhibition of 1–40% at 48 h (mature biofilm). This may be due to carbohydrates dissolved by the water acting as nutrients for the bacteria. In contrast, the acetone, MeOH: DCM, and hot water extracts of both plants showed BIA of between 42 and 59%. The leaves of *N. hildebrandtii* (hot water extract) showed stronger inhibition ranging from 59 to 62% at the T0, T4 and T48 biofilm formation stages. Promising activity of the water extracts indicates that *N. hildebrandtii* leaves could possibly be developed into traditional medicinal teas to treat or prevent diarrhoeal episodes if the safety can be confirmed.

Additionally, at 0 h biofilm against *E. coli*, *N. hildebrandtii* and *N. buchananii* acetone and MeOH: DCM extracts had good inhibition (Fig. 2b). Anti-biofilm activity of the water extracts of both plants showed enhancement. For the 4 h biofilm, *N. hildebrandtii* MeOH: DCM extract had poor BIA of 22% while all other extracts showed enhancement. The 24 h biofilm showed that no extract had an inhibitory effect on the attachment of *E. coli*, meaning that all extracts were enhancing growth at this time point. In the 48 h biofilm *N. hildebrandtii* MeOH: DCM and hot water extracts had poor activity of approximately 0.3–9%. In contrast, the *N. buchananii* acetone leaf extract had good BIA of 55% against *E. coli* (a common diarrhoea-causing pathogen). The compound myricitrin showed excellent BIA of 84% which indicates that it may be responsible for most of the BIA in the crude extract. Furthermore, good anti-biofilm activity of myricitrin against *E. coli* was observed at all time periods (T0, T4, T24 and T48).

At T0, all the extracts had good BIA ranging from 57 to ≥100% against *S.* Typhimurium biofilm (Fig. 2c) besides the *N. hildebrandtii* cold water extract and *N. hildebrandtii* hot water extract which had poor activity and enhancement, respectively. At the 4 h biofilm production, all extracts resulted in enhancement. At T24 the acetone and MeOH: DCM extracts of both plants had BIA of approximately 16–73%. The water extracts of both plants caused enhancement. At T48 the inhibition of the extracts ranged from 8 to 86%. Acetone and MeOH: DCM extracts of both plants and *N. hildebrandtii* cold water extract had poor BIA while *N. hildebrandtii* hot water extract and *N. buchananii* cold and hot water extracts had good activity. Good anti-biofilm activity of myricitrin (above 50%) against *S.* Typhimurium was observed at all time periods (T0, T4, T24 and T48).

The acetone, MeOH: DCM, and hot water extracts of *N. hildebrandtii* and *N. buchananii* acetone and MeOH: DCM extracts had inhibition ranging from approximately 33–50% in the initial attachment stage (0 h biofilm) against *B. cereus* (Fig. 2d). At 4 h (T4) biofilm, *N. hildebrandtii* acetone and MeOH: DCM extracts showed enhancement while other extracts had good activity of approximately 78 to ≥100%. In the 24 h biofilm, *N. hildebrandtii* MeOH: DCM and *N. buchananii* acetone extracts were below 0 while the other extracts had inhibition ranging from 1 to 58%. However, all extracts had poor to very poor activity in the mature biofilm (48 h) and the lack of BIA may be due to the spore-forming activity of this organism as well as the complexity of the biofilm structure. Myricitrin had good activity in all biofilm stages against *B. cereus*.

In the biofilm assay against *E. faecalis* (Fig. 2e), at T0 the acetone and MeOH: DCM extracts of both plant species had good activity whilst the water extracts of both plant species enhanced growth. At 4 h biofilm formation, all extracts showed enhancement. At the maturation and dispersion of the biofilm (24 h and 48 h), there seemed to be a similar effect. The water extracts of both plant species had very good activity of approximately 64- ≥ 100%. Correspondingly, acetone extracts of both plant species had BIA of 26–50% while MeOH: DCM extracts of both plant species had BIA of 1–46%. Generally, at T24 and T48 BIA was good compared to the planktonic and attachment stages. This means that the plant extracts have the potential to overcome resistance by inhibiting biofilm formation. Myricitrin had good activity in all biofilm stages against *E. faecalis.*

The T0 biofilm against *S. aureus* (Fig. 2f) showed that the acetone and MeOH: DCM extracts of both plant species had good BIA whilst the water extracts of both plant species enhanced biofilm development. At 4 h and 24 h biofilm phases all crude extracts showed enhancement. At T48 extracts showed inhibition ranging 19–82% where *N. hildebrandtii* acetone and hot-water extracts had poor BIA of ~ 37 and 19%, respectively. Myricitrin had good activity in all biofilm stages against *S. aureus*.

## Discussion

The antimicrobial activity of myricitrin complements the findings of the activity of the crude extract and fractions of *N. buchananii*. However, the compound had much lower antimicrobial activity than the crude extract, fractions and sub-fractions. The separation of compounds therefore leads to a decrease in activity, and increased toxicity [22]. Interestingly, the crude extracts prepared with organic solvents had very good antimicrobial activity against *P. aeruginosa* with MIC of 20 μg/ml [14] but the compound had low activity of 250 μg/ml in this study. In contrast, Aderogba and co-workers reported myricetin-3-*O*-rhamnoside (isolated from *Croton menyharthii*) to be active against *E. coli* and *S. aureus* at a much higher MIC of 250 μg/ml [23]. This indicates that the possible application of myricitrin would be more for its low toxicity than its moderate activity. According to Wagner and Ulrich-Merzenich (2009), synergistic effects occur if the constituents of an extract affect different targets or interact with one another to improve the solubility, thereby enhancing the bioavailability of one or several substances of an extract [24]. It is also possible that the highly active compounds were not isolated or were inactivated during the isolation procedure.

It has been established that pure drugs isolated from plants rarely have the same degree of activity as the crude extract at comparable concentrations or doses of the active component [25]. This may be due to crude extracts generally consisting of several compounds which may act synergistically with one another [21, 26]. Most traditional health practitioners recommend the use of plants as a whole rather than the isolated compounds because of this synergistic effect [27]. Our results recommend the use of *N. buchananii* leaf extract or fractions rather than a single compound for the treatment of diarrhoea as an antimicrobial agent.

**Fig. 1:**
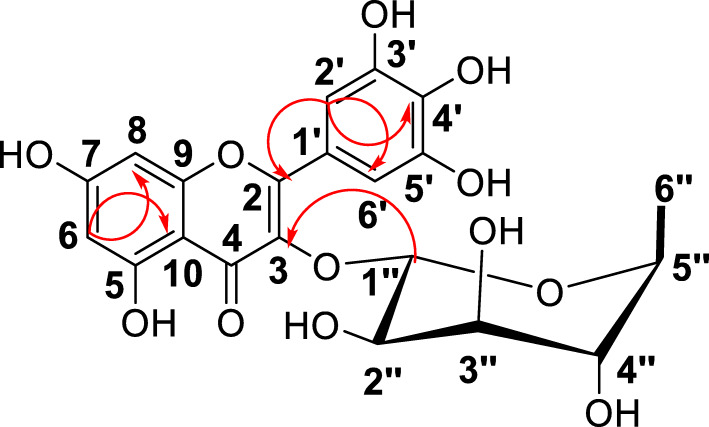
HMBC (arrows) and ^1^H:^1^H COSY (bold) correlations of myricitrin

The structure of myricitrin was elucidated by means of 2D spectroscopic analysis including COSY, HMQC, and HMBC (Fig. 1). A search in the Dictionary of Natural Products [28] and comparing the spectroscopic data with the literature confirmed the structure as the flavonoid myricetin-3-*o*-rhamnoside (myricitrin) [29]. This is the first report of isolation of the flavonoid myricetin-3-*o*-rhamnoside (myricitrin) from *N. buchananii*. Myricitrin has previously been isolated from *Croton menyharthii*, *Euphorbia davidii*, *Myrtus communis*, *Pistacia chinensis*, *Plumbago europaea*, *Santaloides afzelli* and *Searsia chirindensis*, and the compound is known for its antimicrobial, antioxidant, antigenotoxic, anti-inflammatory and antifibrotic activity [19, 30–36].

Furthermore, good anti-biofilm activity of the compound myricitrin against *P. aeruginosa* was observed at all time periods (T0, T4 and T24) with the exception of T48. Infections caused by *P. aeruginosa* may be serious and life-threatening and hard to control by most antibiotics due to its cell wall properties and ability to form biofilms [37]. Additionally, myricitrin had good anti-biofilm activity against all the pathogenic strains known to cause diarrhoea that we investigated. Lopes et al. (2017) studied the inhibitory effects of the glycone myricitrin and the aglycone flavonoid myricetin, in addition to other flavonoids on biofilm formation by *S. aureus* RN4220 and *S. aureus* SA1199B, which are able to overexpress the msrA and norA efflux protein genes. The authors discovered that aglycone myricetin inhibited biofilm formation of *S. aureus* RN4220 and *S. aureus* SA1199B by MBIC_50_ values of 1 and 32 μg/ml, respectively. While myricitrin exhibited MBIC_50_ of 128 μg/ml against *S. aureus* RN4220 and did not show biofilm inhibitory effect against *S. aureus* SA1199B [38]. The authors indicated that myricitrin, myricetin, and other studied flavonoids had weak inhibition on the growth of *S. aureus* strains that overexpress efflux protein genes. While sub-MICs of both myricitrin, myricetin, and other flavonoids showed inhibitory potential on biofilm formation in these strains.

The antibiofilm potential of flavonoids isolated from plants has been reported [39–41].

Moreover, recent studies investigated the inhibitory effects of plant extracts on biofilm formation and the results were in agreement with our findings, for instance, Wijesundara and Rupasinghe (2019) investigated 14 ethanol extracts of selected medicinal plants on bacterial growth and biofilm formation of *Streptococcus pyogenes*. The authors found that the most effective extracts had MIC and MBC of 62.5 μg/mL and 125 μg/mL, respectively, while the MBIC (minimum biofilm inhibitory concentration) ranged from 31.5–250 μg/mL [42].

Alam et al. (2020) evaluated anti-biofilm activity of different extracts of traditionally used plants of Himalayan region of Pakistan against infectious pathogen-*Pseudomonas aeruginosa* PAO1. The authors suggested that various solvent extracts showed different activity against the *P. aeruginosa* PAO1 biofilm. It was found that the 1% methanolic extract of *Bergenia ciliata* exhibited 80% inhibitory effect on biofilm formation without affecting the growth of the bacterium. Interestingly, the authors indicated a significant correlation in the methanolic extract between flavonoid content and anti-biofilm potential, which confirms the inhibitory effects of flavonoids against *P. aeruginosa* (PAO1) [43].

According to Sandasi et al. (2009) the enhanced biofilm development may be due to the presence of certain compounds within the crude plant extracts that provide a conditioning film promoting microbial adhesion [17]. Both plant species had anti-biofilm activity at the MIC value obtained against the planktonic stage though *N. buchananii* had more activity. The inhibition of biofilm formation may be related to the ability of this compound to inactivate microbial adhesins [44]. However, for commercial purposes it would be logical to use the active extracts. Moreover, leaves are easily accessible and traditional healers can prepare formulations using either cold water or boiling water. The dried, ground leaf powder may be soaked in hot or cold water, and the water extract can be used traditionally to relieve symptoms associated with diarrhoea. There is potential of using this plant as a tea to treat diarrhoea and related gastrointestinal conditions since the boiling hot water extract had more anti-biofilm effect than the cold water extract. The dried ground leaf powder of this plant species may be used as tea to alleviate diarrhoeal symptoms, but in vivo studies need to be conducted to confirm the useful antidiarrhoeal efficacy and lack of toxicity of this extract. The water extracts had poor antibacterial activity with MIC values above 100 μg/ml but had good anti-biofilm activity. This may mean that the low antibacterial activity during the planktonic stage of growth does not limit the potential of the extract to inhibit or prevent biofilm formation. As reported in Motlhatlego et al. (2018) [14] *N. buchananii* acetone and MeOH: DCM leaf extracts had good antibacterial activity of 20–80 μg/ml against *B. cereus*, *P. aeruginosa*, *S.* Typhimurium and *S. aureus*. However, anti-biofilm activity at 48 h was only observed against *S. aureus* and *S.* Typhimurium and not the other two organisms.

Extracts of these plant species had stronger inhibitory effects against Gram-positive than Gram-negative bacteria and the most sensitive bacterial strains were *E. faecalis* and *S. aureus*. This may be because Gram-positive bacteria are more susceptible to the action of the extracts that contain flavonoids such as myricitrin [45]. Moreover, Gram-negative bacteria have a different cell wall which decreases uptake. Ciprofloxacin had very good anti-biofilm activity against all six bacteria tested in this study. Shafiei et al. (2014) emphasized the importance of determining how conventional antibiotics affect the ability of anti-biofilm agents to control biofilm perpetuation [46].

## Conclusion

**Fig. 2:**
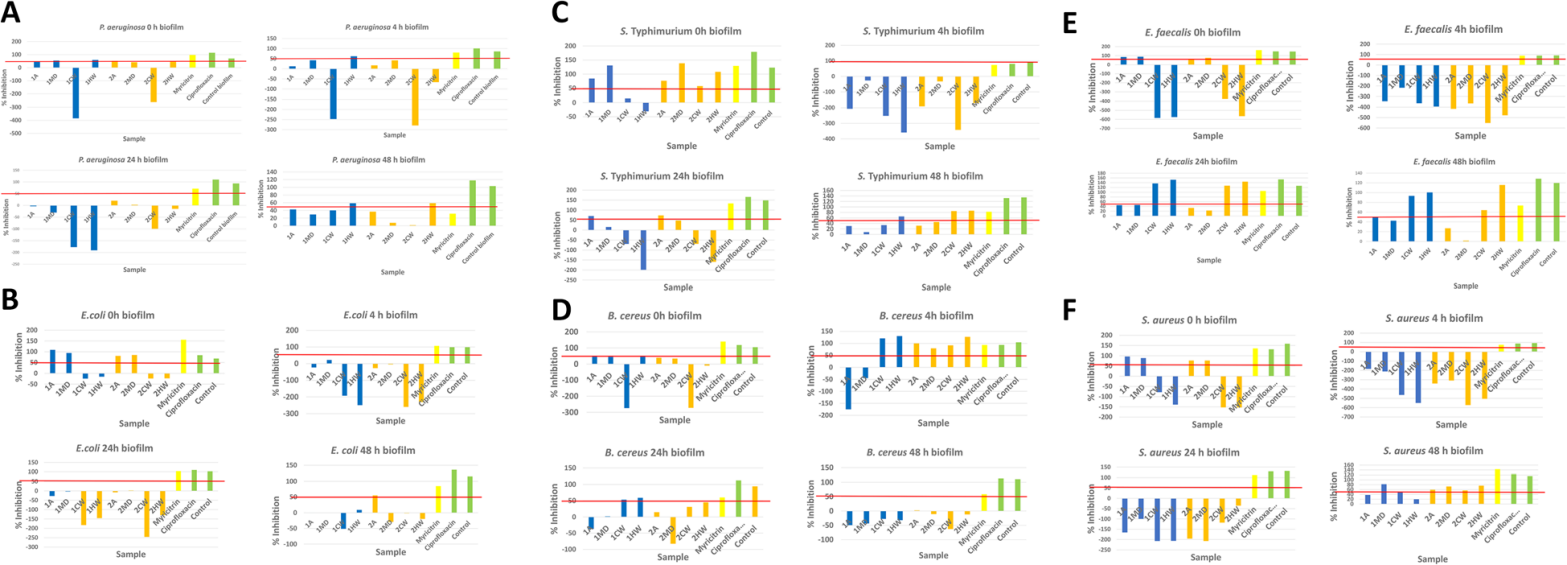
0, 4, 24 and 48-h biofilm inhibition of *Newtonia hildebrandtii* and *Newtonia buchananii* extracts against bacterial strains known to cause diarrhoea. *N. hildebrandtii* acetone leaf extract (1A), *N. hildebrandtii* Methanol-DCM leaf extract (1MD), *N. hildebrandtii* cold-water leaf extract (1CW), *N. hildebrandtii* hot water leaf extract (1HW), *N. buchananii* acetone leaf extract (2A), *N. buchananii* MeOH: DCM leaf extract (2MD), *N. buchananii* cold-water leaf extract (2CW), *N. buchananii* hot water leaf extract (2HW)

This study demonstrated the therapeutic significance of the flavonoid myricetin-3-*o*-rhamnoside (myricitrin), isolated for the first time from *Newtonia buchananii*, a plant used for diarrhoea, against bacterial pathogens. This flavonoid had moderate antibacterial activity against the planktonic forms of *E. coli*, *B. cereus* and *S. aureus* with MIC = 62.5 μg/ml. This study also suggested that the leaf cold and hot water extracts of *N. buchananii* are a potential source of natural antibiofilm agents for gastrointestinal disorders, particularly diarrhoea. Biofilm formation remains a worldwide public health concern and research on the efficacy of novel molecules to prevent this formation is a priority. The antibiofilm potential of myricitrin against *P. aeruginosa*, *E. coli*, *S.* Typhimurium, *E. faecalis*, *S. aureus* and *B. cereus* is a promising tool for reducing microbial colonization on surfaces and epithelial mucosa leading to gastrointestinal infections, particularly diarrhoea. Myricitrin was effective in inhibiting biofilm formation of *S. aureus* strains. In this study myricitrin showed a good antibiofilm dispersal effect against *S. aureus* (Fig. 2f). To the best of our knowledge this is the first report of BIA of *N. hildebrandtii* and *N. buchananii*. The development of potential antibiofilm strategies is of substantial interest. The rational next step would be to determine if there is increased synergistic effect if the two species are respectively combined with ciprofloxacin. A combination of *Newtonia* leaf extracts with ciprofloxacin may possibly offer a novel strategy to effectively control diarrhoeal biofilm-based infections.

## Data Availability

Not applicable.

## Abbreviations

MIC: Minimum inhibitory concentration
MBC: Minimum bactericidal concentrations
MBIC: Minimum biofilm inhibitory concentration
MeOH: Methanol
DCM: Dichloromethane
MTT: 3-(4,5-dimethylthiazol-2-yl)-2,5-diphenyltetrazolium bromide
INT: *p*-iodonitrotetrazolium violet
SI: Selectivity index
LC_50_: 50% lethal concentration
PBS: Phosphate-buffered saline
DMSO: Dimethylsulfoxide
BIA: Biofilm inhibitory activity
